# From loneliness to depression: A longitudinal diagnostic study among Norwegian university students

**DOI:** 10.1007/s00127-025-02989-y

**Published:** 2025-09-17

**Authors:** Mari Hysing, Keith J. Petrie, Allison G. Harvey, Børge Sivertsen

**Affiliations:** 1 Department of Psychosocial Science, Faculty of Psychology, University of Bergen, Bergen, Norway; 2 Department of Psychological Medicine, University of Auckland, Auckland, New Zealand; 3 Department of Psychology, University of California, Berkeley, USA; 4 Department of Health Promotion, Norwegian Institute of Public Health, Bergen, Norway; 5 Department of Research & Innovation, Helse-Fonna HF, Haugesund, Norway

**Keywords:** Loneliness, Young adults, Depression, Students, Higher education

## Abstract

**Purpose:**

Loneliness is an increasing public health concern among young adults. There is insufficient prior research on the association between loneliness and depressive disorder among students in higher education.

**Methods:**

This prospective population-based cohort study from Norway invited all full-time students aged 18–35 to participate in the 2022 Students’ Health and Wellbeing Study (SHoT). Of the 53,362 respondents, a subgroup of 16,418 students was randomly selected for diagnostic follow-up, with valid data from 10,460 participants. Loneliness was assessed in 2022 using the Three-Item Loneliness Scale (T-ILS) and Major Depressive Episodes (MDE) were assessed in 2023 using a self-administered electronic version of the Composite International Diagnostic Interview, version 5.0 (CIDI 5.0).

**Results:**

A clear dose-response relationship was observed: students in the highest loneliness quintile had a substantially increased risk of MDE one year later. After adjusting for age and baseline anxiety and depression symptoms, the relative risk (RR) for MDE in the highest versus lowest T-ILS quintile was 2.02 (95% CI: 1.58–2.63) for females and 2.64 (95% CI: 1.63–4.49) for males—representing a ~ 70–75% reduction from unadjusted estimates. The overall prevalence of MDE was 21.1% in females and 11.2% in males. Formal interaction analyses indicated a statistically significant multiplicative interaction by sex, but no evidence of additive interaction.

**Conclusions:**

Loneliness is a strong and independent predictor of MDE in young adults, even after accounting for baseline mental health. Targeting loneliness may be important for preventing depression in university populations.

**Supplementary Information:**

The online version contains supplementary material available at10.1007/s00127-025-02989-y.

## Introduction

Loneliness is characterized as a subjective deficiency in meaningful social interactions and has long been associated with adverse health outcomes [1–3]. Recent studies have raised the concern that loneliness may be a public health issue of comparable significance to other established risk factors such as obesity, physical inactivity, and smoking [4, 5], though this comparison has been debated [6].

Of particular concern is the rising incidence of loneliness among university students, a group who are navigating major life transitions and academic stress, often with less direct help from parents for the first time [7]. A meta-analysis spanning studies from 1976 to 2019 indicated a linear annual increase in loneliness among young adults [8]. Moreover, Norwegian data have shown a steady increase in loneliness among university students since 2014. While the level of loneliness peaked during the COVID-related campus lockdowns in 2021, data from 2022 showed that loneliness still remained high compared to pre-pandemic levels [9]. While young adults who are not in education or employment may also experience high levels of loneliness, university students represent a distinct and growing subgroup with unique risk factors and challenges related to academic demands, transitions, and social displacement.

Previous research indicates that loneliness is associated with a range of adverse health outcomes, including mental health problems, particularly depression [10]. Indeed, loneliness and depression are closely interlinked, with studies suggesting a bidirectional relationship [11, 12]. While loneliness may act as a potent risk factor for major depressive disorder (MDD) in young adults [13], depression itself can also contribute to increased feelings of loneliness [14]. This reciprocal influence underscores the need for longitudinal research to clarify the temporal direction of the association. As loneliness levels have increased in recent years, it may be an important contributor to rising rates of mental health problems and depression among young adults [15–17]. MDD, in particular, is a concern, as it is not only one of the most prevalent mental health disorders, but it is a leading cause of disability worldwide [18, 19].

Much of the existing literature focused on self-reported symptoms has utilized brief scales that measure depressed mood or self-reported disorders, rather than formal diagnostic criteria [2, 20, 21]. While this approach captures a broad sense of mental well-being, it may not accurately reflect the prevalence of a clinically diagnosed MDD. Utilizing validated diagnostic tools, such as the Composite International Diagnostic Interview (CIDI), can provide a more precise understanding of the links between loneliness and clinically significant MDD. Moreover, meeting formal diagnostic criteria often indicates a more severe level of impairment, requiring targeted intervention. In the few studies using diagnostic outcomes, a British twin study reported a twofold risk of concurrent Major Depressive Episode (MDE) and loneliness among young adults [22].

Taken together, there is a pressing need for longitudinal studies employing diagnostic methodologies to examine the prospective association between loneliness and depressive disorders among university students. The aim of the present study is to prospectively investigate the association between loneliness and depression among young adults in higher education. Given known sex differences in both loneliness and depression, and the potential for different underlying mechanisms, the study also examines whether the strength of this association varies between male and female students.

## Methods

### Setting and participants

The participants were drawn from the SHOT study (Students’ Health and Wellbeing Study), a nationwide survey focusing on students enrolled in higher education across Norway. Since 2010, four major surveys have been conducted, with the most recent wave conducted in 2022. SHOT2022 comprehensively measured various aspects of health and lifestyle, including psychological distress, suicidality, life satisfaction, loneliness, sleep problems, sexual harassment, pain, physical exercise, alcohol and drug use, as well as demographic and educational parameters. Detailed information about SHOT has been published elsewhere [23]. 

During the survey period, SHOT2022 was distributed electronically through a web-based platform and was open for responses from February 8 to April 19, 2022. All full-time Norwegian students pursuing higher education, both domestically and abroad, were invited to participate. Efforts were made to raise awareness about the study through email, SMS, and information campaigns by numerous welfare organizations and educational institutions. The total number of students was 169,572 (58.4% females), out of which 59,544 students completed the online questionnaires after receiving two reminders. This resulted in a response rate of 35.1%. The response rates across the four health regions in Norway were relatively consistent, ranging from 32.1 to 37.5%, as determined from aggregated data obtained from the Norwegian State Educational Loan Fund. For the current study, a subset of 53,362 students was selected. Inclusion criteria included valid consent for participation in SHOT2022, aged 18–35 years, full-time enrolment in higher education, and completion of key variables relevant to the current analysis (loneliness and CIDI data).

When consenting to participate in the SHOT2022, students were given the option to express their interest in participating in a follow-up study on mental disorders. Out of the total participants, 26,311 students consented to be a part of this follow-up study. To maintain a similar distribution of males and females as in the base study population, a greater number of male students were randomly invited to take part. This resulted in a final invited sample of 16,418 students who were officially registered as students in January 2023. However, the number of male students who consented to being contacted for the follow-up study was relatively lower than females, leading to a higher proportion (70.4%) of females receiving invitations to participate in the CIDI study.

**Fig. 1:**
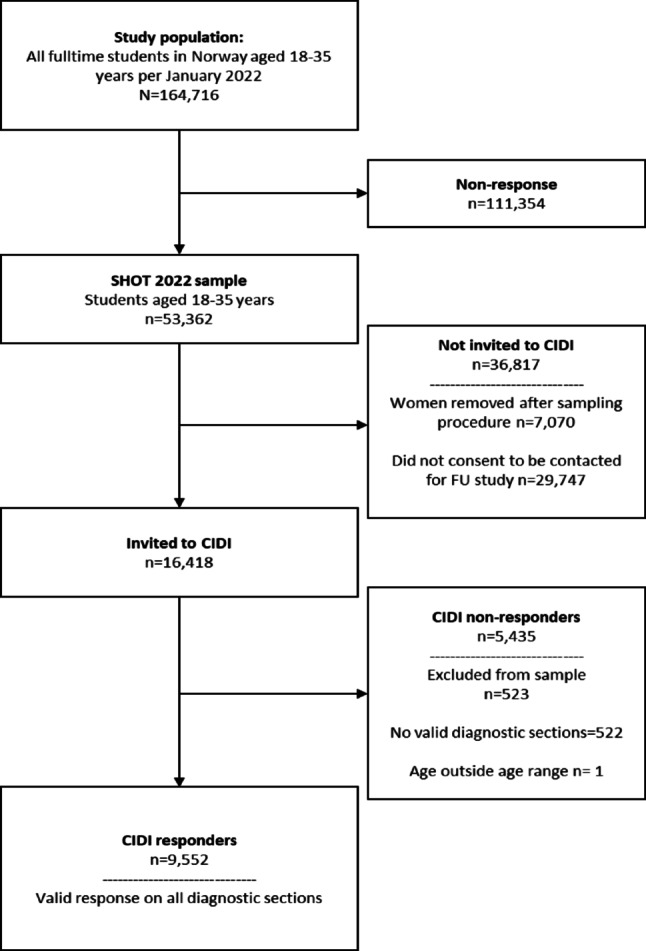
Flowchart of the study participants

A total of 10,460 students provided valid responses on at least one of the CIDI diagnostic sections, resulting in a conditional response rate of 63.7%. The CIDI study took place between January 24 and February 6, 2023, approximately 12 months after the SHOT2022 survey was conducted. More detailed information on the participation process has been published elsewhere [24]. Fig. 1 details the participation process.

### Instruments

#### Sociodemographic information

Participants’ age and sex information was derived from their 11-digit Norwegian national identity numbers. To gather additional background information, the CIDI study was linked with the SHOT2022 study. In SHOT2022, participants were asked about their relationship status, with response options including ‘single,’ ‘boy-/girlfriend,’ ‘cohabitant,’ ‘married,’ or ‘registered partner.’ Participants were also asked about their or their parents’ birthplace, to determine if either the student or their parents were born outside Norway.

#### Loneliness

Loneliness was assessed using an abbreviated version of the widely used UCLA Loneliness Scale, known as the “Three-Item Loneliness Scale (T-ILS)” (Hughes et al., 2004). The T-ILS items (lack of companionship, feeling left out, and isolation) were each rated on a 5-point scale (“never”, “seldom”, “sometimes”, “often”, and “very often”). The T-ILS has displayed satisfactory reliability and both concurrent and discriminant validity (Hughes et al., 2004). Both the individual T-ILS items, as well as a total sum score was used in the current study, the latter was categorized into quintiles. For interaction analyses, the total score was dichotomized at the 80th percentile to identify those with high levels of loneliness. More information about loneliness in the SHOT study has been published elsewhere (Hysing et al., 2020). In addition, SHOT2022 also included a single item assessing to what extent the student felt s/he had enough friends at their place of study, with the response options “I have many friends”, “I have some friends”, “I have few friends”, and “I have no friends”.

#### Mental health problems

Baseline mental health problems in 2022 were assessed using the Hopkins Symptoms Checklist (HSCL-25), a widely used screening tool designed to detect symptoms of anxiety and depression. The HSCL-25 includes a 10-item subscale for anxiety and a 15-item subscale for depression, with each item scored on a 4-point scale ranging from “not at all” [1] to “extremely” [4]. The reference period for the items is the prior two weeks. A prior investigation of the HSCL-25’s factor structure, based on the SHOT2014 dataset, further established that a unidimensional structure, rather than separate subscales for anxiety and depression, exhibits optimal psychometric properties for application to student populations [25]. In this study, we applied sex-specific cutoffs of 1.96 for males and 2.20 for females, as determined in a recent analysis of the SHOT dataset [26]. These updated thresholds were validated against a self-administered version of the Composite International Diagnostic Interview (CIDI). The validation demonstrated that these cutoffs provide an optimal balance between sensitivity and specificity, enhancing the diagnostic precision of the HSCL-25 for use in student populations. To identify individuals with high levels of mental health problems, we dichotomized the HSCL-25 scores based on these cutoffs. Participants scoring above the sex-specific thresholds were categorized as “high scorers,” representing those at elevated risk for significant symptoms of anxiety and/or depression.

#### Mental disorders: the CIDI

A newly developed self-administered electronic version of the Composite International Diagnostic Interview (CIDI), created for the WHO World Mental Health (WMH) Surveys [27] was used for the data-collection [28]. A detailed description of the development of this self-administered version CIDI version has been published elsewhere [24]. In short, CIDI 5.0 is a standardized interview assessing 30-days, 12 months and lifetime prevalence for several mental and substance use disorders according to diagnostic criteria in the Diagnostic and Statistical Manual of Mental Disorders 5th edition (DSM-5) [29]. CIDI 5.0. has good concordance with diagnostic instruments such as the Structured Clinical Interview for DSM-IV (SCID) [30] and Schedules for Clinical Assessment in Neuropsychiatry (SCAN) [31]. The Norwegian version of the CIDI is based on the official Norwegian translation of CIDI 5.0, as described in a previous study protocol publication [32]. 

A current disorder was defined as the presence of MDE during the 30 days before study. We also calculated the 12-month and lifetime prevalence of MDE, and participants fulfilling either of these criteria, but not current mental disorder (*n* = 2,789), were omitted from the statistical analyses, given the presents study’s focus on mental disorders being present after the SHOT2022 data collection. The operationalization of MDE was based on algorithms developed for CIDI 5.0 in the WMH Surveys Initiative.

### Statistical analyses

All analyses were conducted using unweighted data, as the estimates were presented separately for male and female students, and the age and sex distribution of the sample did not differ, or only marginally differed, from that of the base student population. Descriptive and clinical characteristics (age, sex, marital status, country of birth, and loneliness) were calculated for CIDI responders, non-responders, and the total SHOT2022 sample. Statistical comparisons between CIDI responders and non-responders were performed using Chi-squared tests for categorical variables and independent samples t-tests for continuous variables. The prevalence estimates of major depressive episodes (MDE), stratified by levels of loneliness, were subsequently calculated. Poisson regression models with a log link function and robust standard errors were used to calculate effect sizes for dichotomous variables. Results are presented as risk ratios (RR) with 95% confidence intervals, derived from exponentiated coefficients, adjusting for potential confounders. Model 1 adjusted for age, while Model 2 additionally accounted for symptoms of anxiety and depression, as indicated by high scores on the HSCL-25. To assess potential effect modification by sex, we conducted formal interaction analyses on both the multiplicative and additive scales. For the multiplicative interaction, an interaction term (sex × high loneliness) was included in Poisson regression models, adjusting for age and baseline symptoms of anxiety and depression. Additive interaction was evaluated using standard epidemiological measures: the Relative Excess Risk due to Interaction (RERI), the Attributable Proportion due to interaction (AP), and the Synergy Index (S). These were calculated based on risk ratios derived from a four-level combination variable reflecting sex (male/female) and loneliness (high/low, dichotomized at the 80th percentile of the T-ILS score). Evidence of additive interaction is typically indicated by RERI > 0, AP > 0, and S > 1. All analyses were performed using IBM SPSS version 29.

## Results

### Sample characteristics and representativeness

Table 1 provides an overview of the characteristics of students who participated in both the CIDI and SHOT2022 studies. The average age of respondents in the CIDI study was 24 years. The majority of these were Norwegian females, with about half identifying as single. When comparing the demographic characteristics of the CIDI participants to the overall SHOT2022 sample, these were largely similar, except for the sex distribution. The CIDI sample consisted of 70.6% females, whereas the SHOT2022 had 66.4% females. Upon analysing the data provided by students invited to the CIDI study, But who did not participate, the findings were largely consistent with those who did respond, except for loneliness. Students in the CIDI follow-up had a mean T-ILS score of 8.2 (SD = 3.1), which was slightly higher than the score of 8.1 (SD = 3.0; *p* =.04) for students who were invited but chose not to participate. CIDI responders also reported slightly more loneliness than the overall SHOT2022 sample, as detailed in Table 1.

### Overall loneliness and prevalence of MDE

**Fig. 2:**
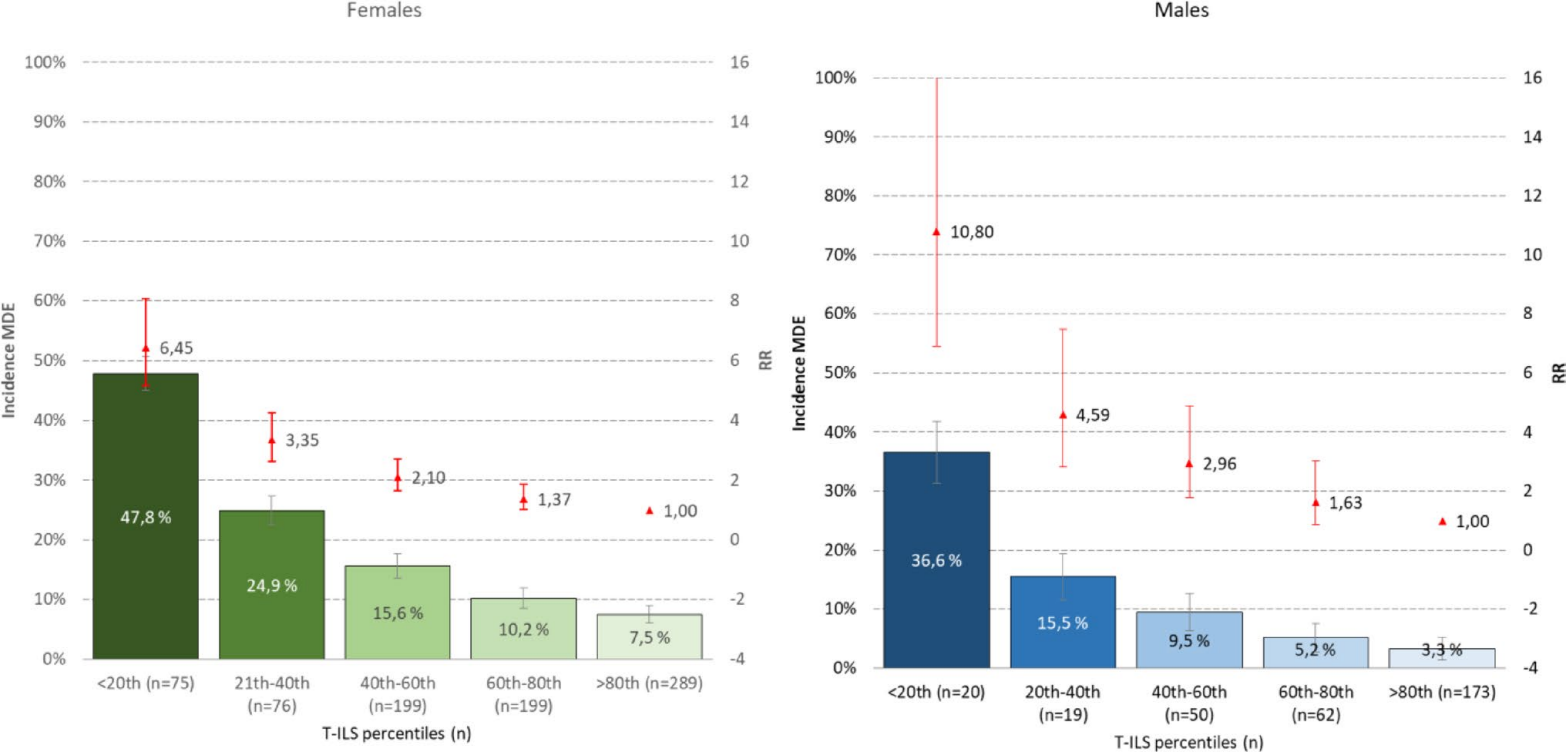
Association between T-ILS total score (quintiles) in SHOT2022 and prevalence of major depressive episode (MDE) in 2023 (CIDI FU-study). Bars represent prevalence (left axis), while the red point estimates (right axis: not on a logarithmic scale) represent age-adjusted relative risk (RR). Error bars represent 95% confidence intervals

Figure 2 illustrates the association between overall loneliness score (in quintiles), measured in the SHOT2022 study and the subsequent prevalence rate of MDE in 2023. A distinct dose-response relationship was observed: as the level of loneliness increased, so did the prevalence of MDE. For example, compared to students who reported the lowest level of loneliness, students in the highest T-ILS quintile had a more than 6-fold increased risk (RR = 6.45, 95% CI: 5.14–8.07) of fulfilling the criteria for an MDE one year later. This trend was evident for both male and female students, although the magnitude of effects were somewhat higher among males, as detailed in Fig. 2.

To assess whether the association between loneliness and MDE differed by sex, formal interaction analyses were conducted. The multiplicative interaction term (sex × high loneliness) was statistically significant (Exp(B) = 0.80, 95% CI: 0.63–1.03, *p* =.027), indicating that the relative risk of MDE associated with high loneliness was lower among females than males. However, additive interaction analyses showed no evidence of effect modification, with a RERI of − 0.11, AP of − 4.8%, and S of 0.92. These findings suggest that while relative risks differ by sex, the absolute risk associated with loneliness is similar, indicating no meaningful interaction on the additive scale.

### Specific loneliness items and prevalence of MDE

**Fig. 3:**
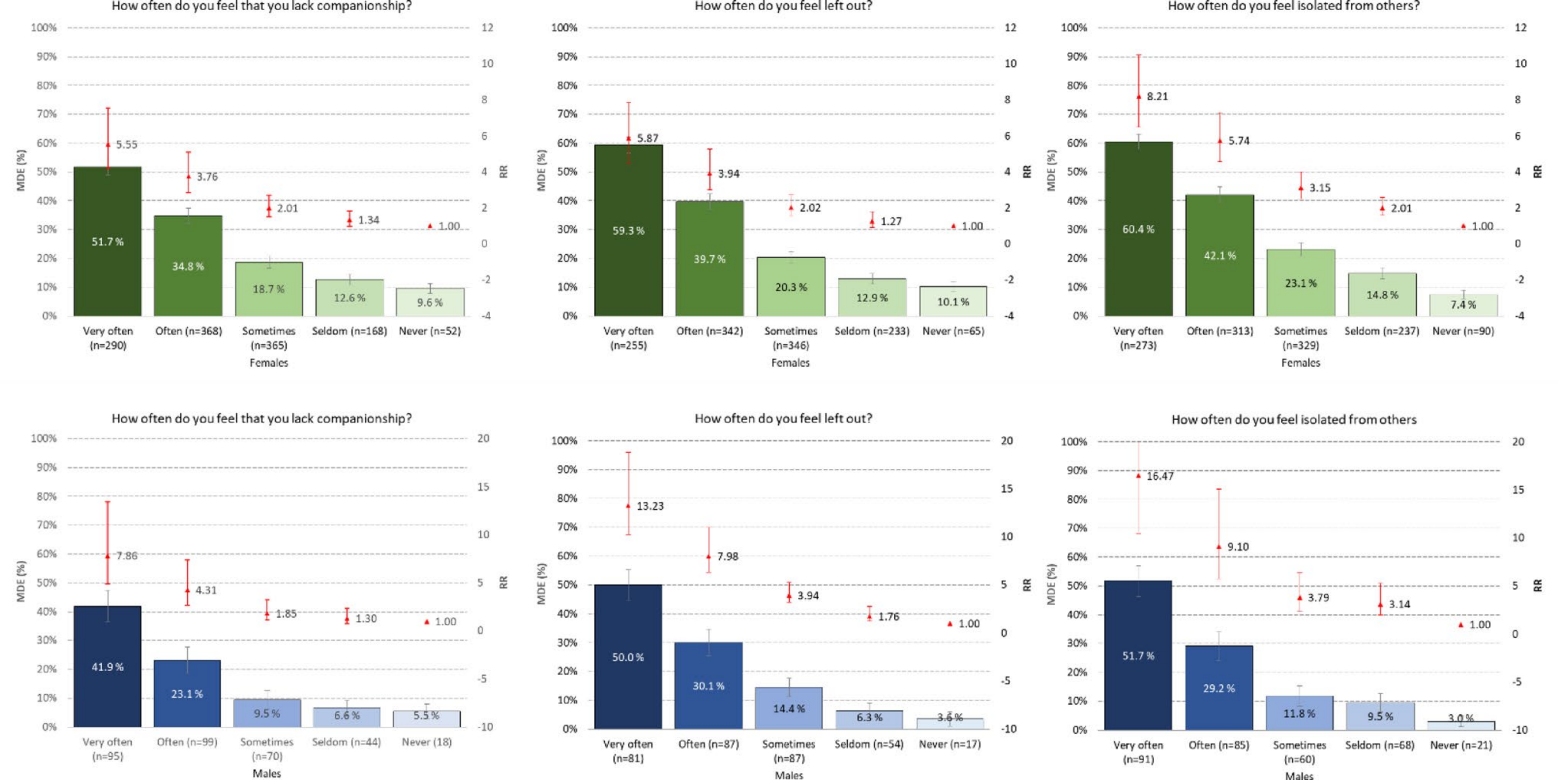
Association between T-ILS items in SHOT2022 and prevalence of major depressive episode (MDE) in 2023 (CIDI FU-study). Bars represent prevalence (left axis), while the red point estimates (right axis: not on a logarithmic scale) represent age-adjusted relative risk (RR). Error bars represent 95% confidence intervals

Figure 3 displays the relationship between the three individual loneliness items and the prevalence of MDE. The general trend was the same as for the overall T-ILS score; the greater the feeling of lacking companionship, feeling left out, and feeling isolated, the higher the risk of MDE one year later. While relative risks were generally higher for males, the absolute differences in prevalence between low and high loneliness groups were greater among females, reflecting their higher baseline rates of MDE. For example, among men who reported never feeling isolated, 3% had an MDE, compared to 52% of those who very often felt isolated (RR = 16.2, 95% CI: 10.4–25.2). Among women, the corresponding rates were 7.4% versus 60.4%, with a relative risk of 8.26 (95% CI: 6.7–12.2).

### Feeling of having enough friends and prevalence of MDE

**Fig. 4:**
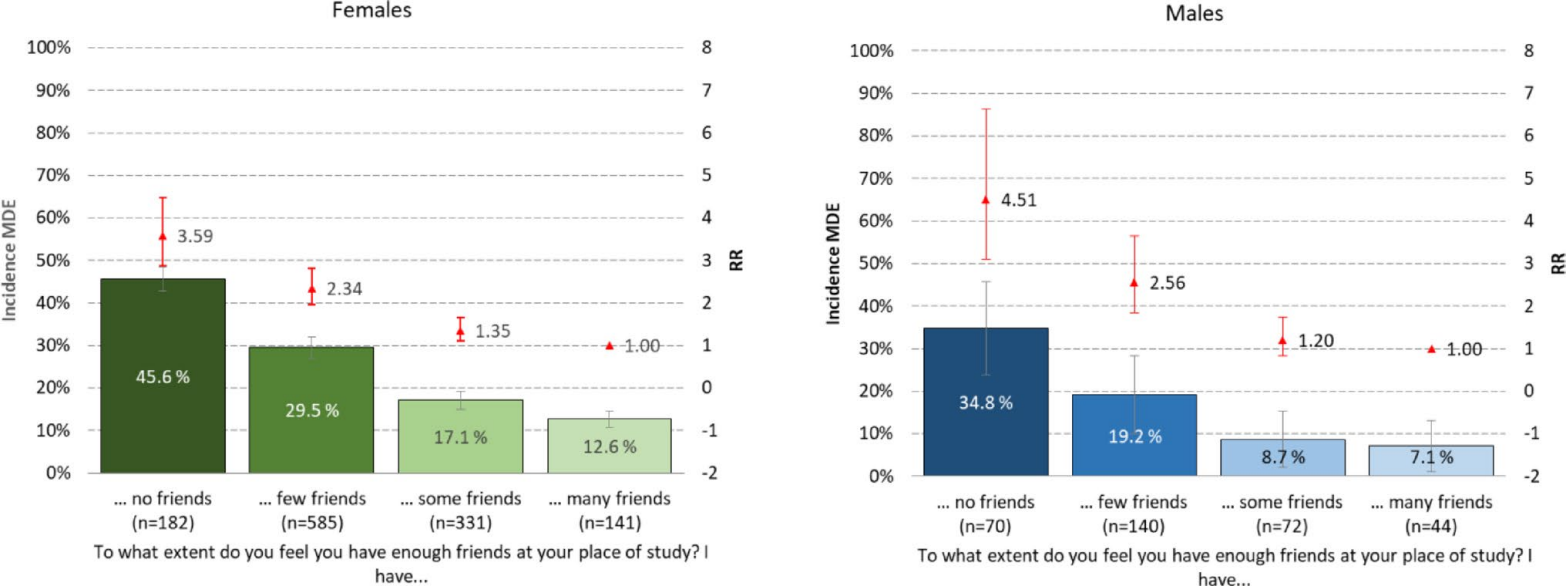
Association between subjective feeling of having enough friends in SHOT2022 and prevalence of major depressive episode (MDE) in 2023 (CIDI FU-study). Bars represent prevalence (left axis), while the red point estimates (right axis: not on a logarithmic scale) represent age-adjusted relative risk (RR). Error bars represent 95% confidence intervals

Figure 4 depicts the relationship between the feeling of having enough friends, and subsequent risk of an MDE. A clear dose-response trend was evident: the stronger the feeling of not having friends, the higher the likelihood of a subsequent depression. For instance, 45.6% of female students who indicated not having any friends had a MDE the following year, in contrast to 12.6% of females who reported having many friends (RR = 3.6, 95% CI: 3.0-4.3). For males, the corresponding rates were 34.8% for those with no friends and 7.1% for those with many friends (RR = 4.5; 95% CI: 3.2–6.3).

### Loneliness and MDE: adjusting for baseline symptoms of anxiety and depression

In Model 2, which adjusted for baseline symptoms of anxiety and depression, risk estimates for major depressive episodes (MDE) associated with loneliness were reduced but remained significant across all measures. A similar dose-response association was observed, where higher levels of loneliness were consistently associated with greater risk of MDE. For example, the relative risk (RR) for MDE among females in the lowest T-ILS percentile (< 20) decreased from 6.42 (95% CI: 5.09–8.23) in Model 1 to 2.02 (95% CI: 1.58–2.63) in Model 2, reflecting approximately a 68% reduction in risk estimates after controlling for baseline anxiety and depression. Among males in the same group, the RR decreased from 10.85 (95% CI: 7.00–17.78) in Model 1 to 2.64 (95% CI: 1.63–4.49), a 76% reduction. These risk estimates are presented in Table 2, which offers the most robust assessment of the association between loneliness and MDE after full covariate adjustment, and should be considered central to interpreting the study’s main hypothesis.

Similar reductions were seen across individual items of T-ILS, with a consistent dose-response relationship. For example, among males who reported “very often” feeling isolated from others, the RR for MDE decreased from 16.47 (95% CI: 10.46–27.18) in Model 1 to 3.51 (95% CI: 2.15–6.00) in Model 2, a 79% reduction. Among females reporting the same, the RR reduced from 8.21 (95% CI: 6.50–10.48) to 2.28 (95% CI: 1.77–2.95), a 72% reduction. These results suggest that loneliness remains a significant and independent predictor of MDE even after accounting for baseline mental health.

To further assess the robustness of this association, we conducted a sensitivity analysis stratifying the sample by baseline HSCL-25 caseness. The dose-response relationship between loneliness and MDE remained evident in both groups, though the strength of the association was attenuated among students with elevated symptoms at baseline. Among those below the HSCL cutoff, students in the highest T-ILS percentile group (> 80) had a 3.74-fold increased risk of MDE (adjusted RR = 3.74, 95% CI: 2.66–5.28), while the corresponding risk among those above the cutoff was 1.61 (95% CI: 1.18–2.27) (see Supplementary Table 1). A formal interaction analysis confirmed this pattern, with a statistically significant interaction term (T-ILS × HSCL-high: adjusted RR = 0.79, 95% CI: 0.71–0.87, *p* <.001), indicating that the relative impact of loneliness on later depression was weaker among those with elevated baseline symptoms.

## Discussion

This study examined the relationship between loneliness and subsequent depression among university students, using data from the national SHOT study and follow-up diagnostic assessments via the CIDI 5.0. Results showed a strong dose-response relationship: students reporting higher levels of loneliness in 2022 were significantly more likely to experience an MDE one year later. This association was evident in both sexes but appeared stronger in men. Students in the highest loneliness quintile had more than a sixfold increased risk of MDE compared to those in the lowest quintile. All aspects of loneliness—including feeling left out, lacking companionship, feeling isolated, and not having enough friends—predicted increased risk, underscoring its central role in student mental health.

Adjusted analyses accounting for baseline anxiety and depression symptoms confirmed the robustness of the association, although relative risks were notably reduced. Sensitivity analyses stratified by HSCL caseness further supported this interpretation: the association persisted in both groups but was stronger among students without elevated baseline symptoms. A statistically significant interaction term also indicated that the relative risk of MDE was modestly attenuated among those already experiencing anxiety or depression.

The current study builds on previous research that has found associations between loneliness and depressive symptoms by incorporating rigorous diagnostic criteria for MDE, validated against the CIDI, which includes assessments of functional impairment, disability, symptom duration, and intensity. A previous study, which examined a broader age range (mean age 54), also reported a consistent, graded association between loneliness and depression, with stronger effects in younger age groups [33]. Our findings reinforce this evidence by demonstrating consistent associations across multiple loneliness indicators and by employing a prospective design with diagnostic outcomes.

While women reported higher levels of both loneliness and MDE, the association between loneliness and depression was evident across both sexes. Notably, formal interaction analyses revealed a statistically significant multiplicative interaction, indicating that the relative risk of MDE associated with loneliness was higher among men. However, additive interaction was not observed, suggesting that the absolute increase in risk was comparable between sexes, despite differences in relative risk. This supports the interpretation that men may be more sensitive to the depressive effects of loneliness, even though they report loneliness less frequently. Taken together, these findings underscore the importance of considering both relative and absolute risk in understanding the sex-specific public health burden of loneliness. Women may still bear a greater burden of disease overall due to their higher prevalence of both loneliness and depression.

A key strength of the current study is its large sample size and recruitment from higher education institutions across Norway. However, the 35% response rate represents a limitation and raises the possibility of non-response bias. Although we were able to compare participants on key indicators like sex and age, we lack detailed information about the mental health status of non-respondents. Still, comparisons indicate that participants in the follow-up diagnostic study had only slightly higher levels of loneliness and HSCL-25 symptoms than the full SHoT cohort. Importantly, the primary aim of the study was to examine associations rather than to estimate prevalence, and such associations are generally less affected by non-response bias [34].

Another limitation is the 12-month follow-up period, which may be relatively short for capturing the full course of depression development. The design also precludes examination of potential reciprocal associations between loneliness and depression over time. Additionally, as MDE onset may have occurred before the SHoT assessment, we cannot fully rule out reverse causality. However, our adjusted models—which controlled for baseline symptoms of anxiety and depression—suggest that loneliness contributes to the development of subsequent MDE. While this supports a directional effect from loneliness to depression, the relationship may also operate in both directions. These findings are consistent with prior studies indicating a bidirectional link between loneliness and depression [35], including a recent Mendelian randomization study using genome-wide association data, which found genetically informed evidence that loneliness increases the risk of major depression, and vice versa [36]. These bidirectional effects remained robust across multiple large samples and sensitivity analyses, supporting the possibility of reciprocal causal links between the two constructs. This further underscores the elevated vulnerability of young adults—especially in the wake of the COVID-19 pandemic [37].

While awaiting replication, the current findings highlight loneliness as a potential target for mental health promotion among young adults. Although causal conclusions cannot be drawn, reducing loneliness may represent one pathway to improving emotional well-being and potentially mitigating the risk of depression. When planning educational formats—such as decisions between digital and physical teaching methods—the potential risk of loneliness should be considered.

While some interventions aimed at increasing social connectedness have shown promise, the overall effectiveness remains mixed (see [38]). Moreover, it is important to distinguish between loneliness—a subjective experience of unmet social needs—and social isolation, which reflects an objective lack of social contact or participation. Although loneliness and social isolation are often used interchangeably, they reflect distinct constructs. Loneliness refers to a perceived lack of meaningful connection, while social isolation typically reflects an objective lack of social contact or participation [39]. In our study, all items—including ‘feeling isolated from others’—reflect subjective loneliness. Notably, this item showed the strongest association with MDE, underscoring the relevance of perceived isolation as a mental health risk factor.

Thus, interventions focused solely on promoting social contact may not be sufficient to reduce loneliness or prevent depression. A broader range of approaches may be needed, including psychological interventions that directly address maladaptive social cognitions [40, 41]. Additionally, universal prevention strategies—such as fostering inclusive campus environments and reducing structural barriers to participation—could play a key role in reducing loneliness. Finally, our findings underscore the need to tailor interventions to specific subgroups, particularly men, who may be more vulnerable to the mental health consequences of loneliness despite reporting it less often.

In conclusion, this large prospective study demonstrates a robust dose-response association between loneliness and the risk of MDE among university students, even after adjusting for baseline mental health symptoms. The association was observed across both sexes and all dimensions of loneliness and remained consistent in sensitivity and interaction analyses. While the relative risk appeared stronger among men, the overall burden of loneliness and depression remains substantial for both sexes. These findings highlight loneliness as a significant and modifiable risk factor for depression in young adults and underscore the importance of addressing loneliness in future mental health prevention strategies in higher education settings.

**Table 1:** Sociodemographic and loneliness in 2022 of the CIDI responders, CIDI non-responders and the overall SHOT2022 sample

Characteristic	CIDI responders (*n* = 10,460)	CIDI non-responders (*n* = 5,958)	*p*-value^#^	SHOT2022* (*n* = 53,362)	*p*-value^#^
Age, mean (SD)	24.0 (3.3)	24.0 (3.2)	0.24	24.0 (1.9)	0.14
Sex, % (n)			0.97		< 0.001
Women	70.6 (7,386)	70.4 (4,196)		66.4 (35,423)	
Men	29.4 (3,074)	29.6 (1,762)		33.6 (17,939)	
Marital status, % (n)			0.16		0.70
Single	51.2 (5,359)	50.3 (2,994)		51.0 (27,197)	
Boy-/girlfriend	22.4 (2,343)	23.7 (1,414)		22.8 (12,152)	
Cohabitant	22.7 (2,375)	22.5 (1,340)		22.6 (12,058)	
Married/registered partner	3.3 (345)	3.0 (178)		3.1 (1,667)	
*Missing*	*0.4 (38)*	*0.5* (32)		*0.5 (288)*	
Self and/or parent(s) born abroad, % (n)			0.17		0.34
Born in Norway	81.2 (8,491)	80.4 (4,792)		80.1 (43,052)	
Born outside Norway	10.0 (1043)	10.9 (651)		10.4 (5,541)	
*Missing*	*8.9 (926)*	*8.6* (515)		*8.9 (4*,*769)*	
Loneliness (T-ILS), Mean (SD) ^$^	8.2 (3.1)	8.1 (3.0)	0.04	8.1 (3.0)	0.01
*Missing*,* % (n)*	*0.9 (89)*	*1.4 (95)*		*1.5 (806)*	

**Table 2:** Association between loneliness in SHOT2022 and risk of major depressive episode (MDE) in 2023 (CIDI FU-study). model 1: adjusting for age; model 2: additional adjustment for baseline symptoms of anxiety and depression

	Females				Males			
	Model 1		Model 2		Model 1		Model 2	
	RR	(95% CI)	RR	(95% CI)	RR	(95% CI)	RR	(95% CI)
T-ILS percentiles								
<20	6.42	(5.09–8.23)	2.02	(1.58–2.63)	10.85	(7.00–17.78)	2.64	(1.63–4.49)
21–40	3.35	(2.62–4.35)	1.67	(1.29–2.19)	4.65	(2.86–7.89)	1.98	(1.19–3.42)
41–60	2.09	(1.61–2.75)	1.43	(1.10–1.88)	2.96	(1.79–5.08)	1.78	(1.07–3.09)
61–80	1.37	(0.99–1.88)	1.14	(0.83–1.58)	1.63	(0.87–3.07)	1.29	(0.68–2.43)
>80	1.00		1.00		1.00		1.00	
How often do you feel that you lack companionship?								
Very often	5.55	(4.17–7.54)	1.74	(1.29–2.38)	7.86	(4.87–13.43)	2.00	(1.21–3.47)
Often	3.76	(2.84–5.09)	1.66	(1.24–2.26)	4.31	(2.68–7.35)	1.77	(1.08–3.05)
Sometimes	2.01	(1.52–2.72)	1.34	(1.01–1.81)	1.85	(1.13–3.2)	1.20	(0.73–2.08)
Seldom	1.34	(0.99–1.84)	1.09	(0.80–1.5)	1.30	(0.76–2.3)	1.12	(0.66–1.99)
Never	1.00		1.00		1.00		1.00	
How often do you feel left out?								
Very often	5.87	(4.50–7.76)	1.67	(1.26–2.23)	13.23	(8.05–23.09)	2.78	(1.63–5.00)
Often	3.94	(3.04–5.18)	1.50	(1.15–1.99)	7.98	(4.88–13.89)	2.14	(1.27–3.83)
Sometimes	2.02	(1.56–2.66)	1.23	(0.95–1.62)	3.94	(2.41–6.85)	1.83	(1.10–3.23)
Seldom	1.27	(0.97–1.69)	1.07	(0.82–1.42)	1.76	(1.04–3.13)	1.36	(0.81–2.43)
Never	1.00		1.00		1.00		1.00	
How often do you feel isolated from others								
Very often	8.21	(6.50–10.48)	2.28	(1.77–2.95)	16.47	(10.46–27.18)	3.51	(2.15–6.00)
Often	5.74	(4.56–7.29)	2.04	(1.60–2.63)	9.10	(5.75–15.06)	2.64	(1.62–4.49)
Sometimes	3.15	(2.50–3.99)	1.66	(1.31–2.12)	3.79	(2.34–6.37)	1.88	(1.14–3.02)
Seldom	2.01	(1.59–2.58)	1.47	(1.16–1.89)	3.14	(1.96–5.25)	2.20	(1.37–3.70)
Never	1.00		1.00		1.00		1.00	
To what extent do you feel you have enough friends at your place of study? I have…								
…no friends	3.59	(2.88–4.48)	1.70	(1.36–2.13)	4.51	(3.10–6.64)	1.75	(1.19–2.60)
…few friends	2.34	(1.95–2.82)	1.43	(1.19–1.73)	2.56	(1.84–3.64)	1.42	(1.01–2.02)
…some friends	1.35	(1.11–1.65)	1.14	(0.94–1.40)	1.20	(0.82–1.75)	0.98	(0.67–1.43)
…many friends	1.00		1.00		1.00		1.00	

## Electronic supplementary material

Below is the link to the electronic supplementary material.

Supplementary Material 1

## Data Availability

Norwegian data protection regulations and GDPR impose restrictions on sharing of individual participant data. However, researchers may gain access to survey participant data by contacting the publication committee (borge.sivertsen@fhi.no). Approval from the Norwegian Regional Committee for Medical and Health Research Ethics (https://helseforskning.etikkom.no) is a pre-requirement for access to the data. The dataset is administrated by the NIPH, and guidelines for access to data are found at https://www.fhi.no/en/more/access-to-data. Analytic codes for the analyses are available upon request to the corresponding author.
