# Supplementary material for: From loneliness to depression: A longitudinal diagnostic study among Norwegian university students
Source: Soc Psychiatry Psychiatr Epidemiol. 2025 Sep 17;61(3):449–59. doi: 10.1007/s00127-025-02989-y (PMC12995926; doi:10.1007/s00127-025-02989-y)
Supplement: Supplementary file 1 — Supplementary Material 1 [file 127_2025_2989_MOESM1_ESM.docx]

| **Supplementary table 1.** Sensitivity and Interaction Analyses for the Association Between Loneliness and Risk of Major Depressive Episode (MDE) | | | |
| --- | --- | --- | --- |
| **Analysis** | **Loneliness level (quintiles)** | **RR (95% CI)** | **p-value** |
| **Sensitivity analysis** |  |  |  |
| HSCL-25 < cutoff | > 80 (vs. <20) | 3.74 (2.66–5.28) | < .001 |
|  | 61–80 | 2.22 (1.59–3.12) | < .001 |
|  | 41–60 | 1.76 (1.27–2.46) | < .001 |
|  | 21–40 | 1.02 (0.67–1.53) | .935 |
| HSCL-25 ≥ cutoff | > 80 (vs. <20) | 1.61 (1.18–2.27) | < .001 |
|  | 61–80 | 1.35 (0.97–1.92) | .028 |
|  | 41–60 | 1.19 (0.84–1.71) | .230 |
|  | 21–40 | 1.20 (0.80–1.81) | .266 |
| Interaction model | T-ILS × HSCL-25 caseness | 0.79 (0.71–0.87) | < .001 |
